# Natural Chalcones in Chinese Materia Medica: Licorice

**DOI:** 10.1155/2020/3821248

**Published:** 2020-03-16

**Authors:** Danni Wang, Jing Liang, Jing Zhang, Yuefei Wang, Xin Chai

**Affiliations:** ^1^Tianjin State Key Laboratory of Modern Chinese Medicine, Tianjin University of Traditional Chinese Medicine, Tianjin 301617, China; ^2^School of Foreign Language, Chengdu University of Traditional Chinese Medicine, Sichuan 611137, China

## Abstract

Licorice is an important Chinese materia medica frequently used in clinical practice, which contains more than 20 triterpenoids and 300 flavonoids. Chalcone, one of the major classes of flavonoid, has a variety of biological activities and is widely distributed in nature. To date, about 42 chalcones have been isolated and identified from licorice. These chalcones play a pivotal role when licorice exerts its pharmacological effects. According to the research reports, these compounds have a wide range of biological activities, containing anticancer, anti-inflammatory, antimicrobial, antioxidative, antiviral, antidiabetic, antidepressive, hepatoprotective activities, and so on. This review aims to summarize structures and biological activities of chalcones from licorice. We hope that this work can provide a theoretical basis for the further studies of chalcones from licorice.

## 1. Introduction

*Glycyrrhiza*, one of the oldest medicinal plants, is often referred to as Chinese licorice, which belongs to the family of Leguminosae [1, 2]. There are 30 species of licorice distributed all over the world. Three *Glycyrrhiza* species including *Glycyrrhiza uralensis* Fisch., *Glycyrrhiza inflata* Bat., and *Glycyrrhiza glabra* L. are prescribed as licorice in the Chinese Pharmacopoeia [3, 4]. Licorice, derived from the dried roots and rhizomes of genus *Glycyrrhiza*, is called “gancao” in China and was firstly recorded in Shennong's Classic of Materia Medica around 2100 BC [5]. It is widely distributed in many provinces of China, such as Xinjiang, Gansu, Inner Mongolia Autonomous Region, Ningxia, and Shanxi, as well as the Siberian region of Russia [5]. As a kind of crucial Chinese materia medica (CMM), licorice has a great many pharmacological effects and is widely used in clinical practice. Licorice is capable of tonifying qi, invigorating spleen and harmonizing stomach, clearing heat, detoxifying, reducing phlegm, relieving cough, alleviating pain, etc. [3, 6]. Licorice, which not only has significant efficacy but also has mild and nontoxic properties, can reconcile plenty of CMMs. Therefore, it can be combined with various CMMs to form a variety of Chinese herbal compound prescriptions, such as Guizhi Gancao decoction, Shaoyao Gancao decoction, and Xuefu Zhuyu decoction, thus exerting different effects [3].

Licorice is not only a bulk medicine for traditional Chinese medicine but also a major raw material for Mongolian medicine, or an important component of many folk prescriptions. In addition, the chemical composition of licorice is very complicated. To date, more than 20 triterpenoids and 300 flavonoids have been isolated from licorice [4]. The total flavonoids of licorice are a large class of compounds. At present, many flavonoids have been isolated from licorice, including dihydroflavones, chalcones, isoflavones, and other types of flavonoids. Chalcones, the vital class of secondary metabolites of plant [7], are not only an important synthetic precursor of flavonoids but also a major form of the natural products [8]. Currently, about 42 chalcones have been isolated from licorice. These chalcones play a pivotal role when licorice exerts its pharmacological effects. Numerous studies have indicated that these bioactivities include anticancer [9], anti-inflammatory [10], antimicrobial, antiviral [5], antioxidative, hepatoprotective [11], antidiabetic [12], antidepressant activities [13], and so on.

Chalcone compounds and their derivatives are important organic synthesis intermediates. Highlighting the broad range of biological activities of chalcones in numerous reports has caused extensive attention. In recent years, people have increasingly studied the components and activities of chalcones in licorice. This work aims to sum up the structures and pharmacological activities of chalcones that have been identified from licorice. This review might provide some dependable experiment gist and a theoretical basis for further research on chalcones in licorice.

## 2. The Structures of Chalcones in Licorice

**Figure 1:**
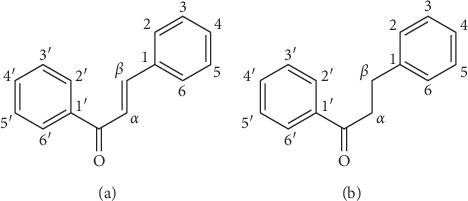
Basic framework of chalcone and dihydrochalcone.

Chalcones are 1,3-diphenyl-2-propene-1-ones, in which two aromatic rings named as A and B ring are linked by a three-carbon *α*,*β*-unsaturated carbonyl system [7]. Chalcones belong to the group of flavonoids, including chalcones and dihydrochalcones (Figure 1). They are not only widespread in nature but also obtained by biosynthesis and chemical synthesis. Since 1988, 42 chalcones including 33 chalcones and 9 dihydrochalcones have been reported in licorice, which were proved to possess a large number of biological activities.

To date, many researchers have analyzed the ingredients in licorice with a variety of methods. For example, based on ultra-performance liquid chromatography-tandem mass spectrometry (UPLC-MS), Jiang et al. detected 19 components in licorice, including isoliquiritin apioside, neoisoliquiritin, and licochalcone A as chalcones [14]. Yin et al. used ultraperformance liquid chromatography-electrospray ionization-quadrupole-time of flight mass spectrometry (UPLC-ESI-Q-TOF-MS) for the rapid analysis of the four chalcones, namely, isoliquiritigenin, isoliquiritin, neoisoliquiritin, and licuraside [15]. By introducing a newly developed high-performance thin-layer chromatography (HPTLC) method, Liu et al. identified several chalcones including isoliquiritin apioside, isoliquiritigenin, isoliquiritin, and so on [16]. Li et al. employed UHPLC-MS to quantify 14 licorice components including five chalcones [17].

**Figure 2:**
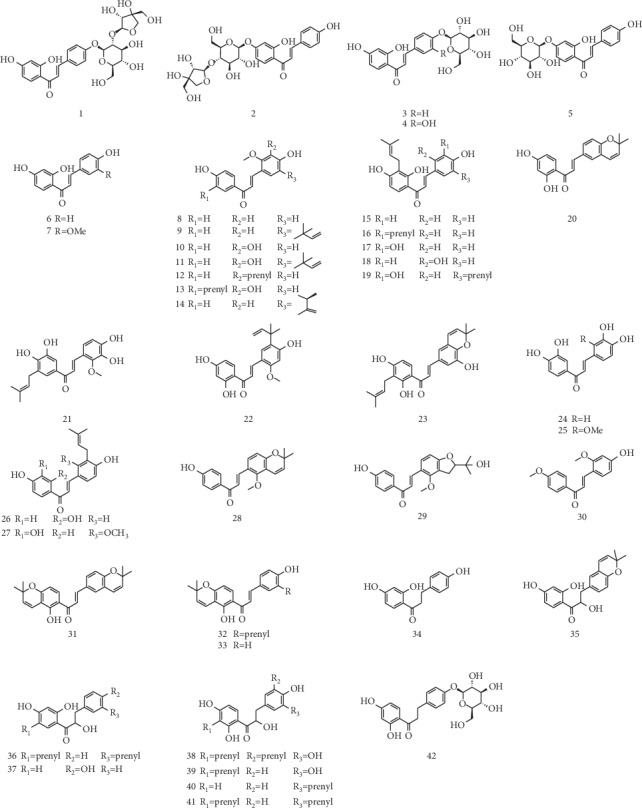
The structures of chalcones from licorice.

To date, about 42 chalcones in licorice have been reported (Table 1). The structures of these compounds were shown in Figure 2.

## 3. The Biological Activities of Chalcones in Licorice

**Figure 3:**
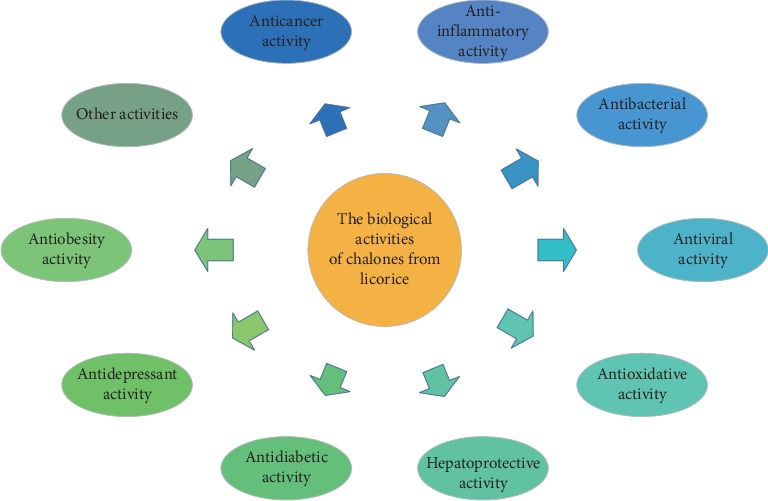
The biological activities of chalcones from licorice.

Chalcones in licorice have been reported to manifest different biological activities, including anticancer, anti-inflammatory, antibacterial, antiviral, antioxidative, hepatoprotective, antidiabetic, antidepressant, and other activities (Figure 3). The following is the summary of the biological activities of chalcones in licorice (Table 2).

### 3.1. Anticancer Activity

Cancer, the enemy of humanity, seriously endangers human life and health. With the aging of the social population structure, environmental pollution, and the prevalence of unhealthy living behaviors such as smoking and unreasonable diet, the situation of cancer will become more intense in a few years. In recent years, phytochemicals from herbal medicine remain a main source of drug discovery [31]. There is no doubt that chalcones in licorice have great influence on the treatment of cancer. Chalcones have the property of electrophiles that interact with nucleophiles in proteins to inhibit the growth of tumor cells. At present, several chalcones from licorice have been demonstrated to possess anticancer property by induction of cell cycle arrest, inhibition of cancer cell metastasis, apoptosis, and autophagy [114].

Isoliquiritigenin is an impactful cancer chemopreventive agent *in vitro* as well as *in vivo.* According to the reports, isoliquiritigenin was resistant to multiple tumor cells. Recent study suggested that isoliquiritigenin induced apoptosis in A549 non-small-cell lung cancer cells by crosstalk between protein 53 (p53), B-cell lymphoma-2 (Bcl-2) family proteins, caspase cascades, and protein kinase B (Akt) survival pathways [32]. It also caused DNA damage and inhibited ataxia-telangiectasia mutated (ATM) expression leading to G2/M phase arrest and apoptosis in oral squamous cell carcinoma [33]. Isoliquiritigenin induced apoptosis in renal cell carcinoma Caki cells via the generation of ROS, resulting in induction of p53 expression and inhibition of the signal transducer and activator of transcription 3 (STAT3) signaling pathway [34]. Moreover, isoliquiritigenin affected WIF1 gene and downregulated *κ*-catenin signal, resulting in the undergoing arrest of breast cancer cells at the G0/G1 phase [35]. In prostate cancer, it affected expression of protein B1, cyclin-dependent kinase 1 (CDK1), and other related molecules in the G2/M cell cycle of PC-3 and 22RV1 cells [31].

Licochalcone A, a characteristic chalcone isolated from licorice roots [36], was proved to be resistant to many types of cancer cells through different mechanisms. Induction of mitochondrial dysfunction is one of the main pathways leading to apoptosis by licochalcone A. It was found that it induced activation of caspases via the mitochondrial pathway and then mediated its antiproliferative and apoptotic effects of oral squamous cell carcinoma by inhibiting Sp1 and Sp1-mediated signaling pathways [37]. Licochalcone A inhibited glioma cell growth by mediating cell cycle arrest at G0/G1 and G2/M phases and induced cell death by inducing mitochondrial dysfunction [38, 39]. Licochalcone A induced apoptosis of gastric cancer cells via the caspase-dependent mitochondrial pathway and exerted G2 cell cycle arrest through the regulation of G2/M phase checkpoint proteins [40]. Additionally, it handled activation of the LC3-II signaling pathway while inhibiting the phosphatidylinositol 3-kinase (PI3K)/Akt/mammalian target of the rapamycin (mTOR) signaling pathway that promoted autophagy and apoptosis in cells [41]. Licochalcone A caused G2 and late-G1 arrests in androgen-independent PC-3 prostate cancer cells by affecting the expression of proliferating cell nuclear antigen (PCNA), DNA polymerase *δ*, Rb and E2F, cyclins B1 and D1, and so on, which led to apoptosis [42].

Licochalcone B led to S-phase arrest, decreased the expression of cyclin A, CDK1, CDK2 mRNA, Bcl-2, survivin, and cell division cycle 25 (Cdc25A and Cdc25B) proteins, induced downregulation of antiapoptotic proteins (Bid, Bcl-xl, and Mcl-1), and also caused the loss of mitochondrial membrane potential (MMP) [43, 44]. However, it enhanced Bax expression, activated caspase-3, and cleaved poly ADP-ribose polymerase (PARP) protein [44]. In addition, it attenuated migration, adhesion, and invasion of cancer cells accompanied with the downregulated protein expression of MMP-9 and nuclear translocation of nuclear factor-*κ*B (NF-*κ*B) [115].

Similar to licochalcone B, licochalcones C and D induced apoptosis by altering the expression of Bcl-2 family member genes and activating the caspase-mediated cell death signaling pathway [45, 46]. Meanwhile, licochalcone D induced apoptosis through mitochondrial pathway and blocked cell migration and invasion into surrounding tissues by reducing the activity and expression of MMP-2 and MMP-9 [46]. In addition to increasing the expression of proteins (proapoptotic factors, caspase-3, and PARP), suppressing the constitutive NF-*κ*B activation, and downregulating Bcl-2 and Bax, licochalcone E reduced tumor growth and metastasis through inhibiting tube formation of vascular endothelial cells [47–49]. Same as licochalcone D, kanzonol C could inhibit MMP-2 secretion from tumor cells [50]. Unlike the preceding compounds, isobavachalcone induced apoptosis through increasing the expression of tumor necrosis factor-related apoptosis-inducing ligand-receptor 2 (TRAIL-R2) [51].

Echinatin triggered apoptosis of esophageal squamous cell carcinoma by inducing intrinsic and extrinsic apoptosis pathways through ROS- and ER-stress-mediated signaling [52]. Additionally, another study has demonstrated that it inhibited cell proliferation and induced apoptosis by targeting epidermal growth factor receptor (EGFR) and mesenchymal-to-epithelial transition (MET) factor in gefitinib-sensitive and gefitinib-resistant non-small-cell lung cancer (NSCLC) cells [53]. Isoliquiritin apioside, as an antigenotoxic substance, showed obvious anticancer effect by preventing H_2_O_2_- and 4NQO- induced DNA damage [54]. Paratocarpin A showed evident anti-invasive activity on MCF-7/6 mammary carcinoma cells [55]. Apart from the above compounds, 4-hydroxylonchocarpin and kanzonol Y also showed anticancer activity [56–58].

### 3.2. Anti-Inflammatory Activity

The inflammatory reaction is a common clinical pathological process that could be born in various parts of body tissues and organs [116]. According to the duration of inflammation, it is divided into two stages: acute and chronic inflammation [56]. In the more serious inflammatory diseases, especially when the pathogenic microorganisms spread in the body, there is obvious systemic reaction [60]. So far, the treatment of inflammation encounters a lot of difficulties. How to safely and effectively treat inflammation is also an issue that should be solved urgently. As a commonly used CMM for the treatment of inflammation, licorice has notable anti-inflammatory effect. Triterpenes and flavonoids from licorice might cure different types of inflammation, especially chalcones [6]. NF-*κ*B and mitogen-activated protein kinase (MAPK) pathways were proved to be important for chalcones to exert anti-inflammatory activity.

Isoliquiritigenin, a natural chalcone extracted from licorice, has been well studied for its anti-inflammatory activity [117]. Confirmed by cell experiments, isoliquiritigenin inhibited inducible nitric oxide synthase (iNOS) and cyclooxygenase-2 (COX-2) protein expressions and iNOS, COX-2, tumor necrosis factor-*α* (TNF-*α*), and interleukin-6 (IL-6) transcriptions by inhibiting degradation and phosphorylation of inhibitor *κ*B-*α* (I*κ*B*α*) and blocking activation of NF-*κ*B [60]. In other cases, isoliquiritigenin suppressed the receptor activator of nuclear factor-B ligand (RANKL)-induced inflammatory symptom via inhibiting the receptor activator of nuclear factor-B ligand-tumor necrosis factor receptor-associated factor 6 (RANK-TRAF6), MAPK, I*κ*B*α*/NF-*κ*B, and activator protein-1 (AP-1) signaling pathways [61]. Isoliquiritigenin inhibited activation of NF-*κ*B and formation of lipopolysaccharide (LPS)-induced toll-like receptor 4/myeloid differentiation protein 2 (TLR4/MD-2) complexes, which further led to suppressing LPS-induced activation of signaling cascades [62]. In addition, isoliquiritigenin and isoliquiritin, to a certain extent, could mediate anti-inflammatory responses of LPS-induced macrophage activation via increasing heme oxygenase-1 (HO-1) and nuclear factor erythroid 2-related factor 2 (Nrf2) expression and inhibiting I*κ*B*α* phosphorylation and degradation [59]. Additionally, isoliquiritigenin prevented nonsteroidal anti-inflammatory drug (NSAID)-induced small intestinal damage by the inhibition of NOD-like receptor (NLR) family and pyrin domain containing 3 (NLRP3) inflammasome activation [63] and ameliorated the dextran sulfate sodium (DSS)-induced colitis through inhibiting MAPK pathway [64]. It also has been demonstrated that isoliquiritigenin exerted anti-inflammatory effect through inhibiting eotaxin-1 secretion and suppressing IL-1, IL-8, and caspase-1 production [65–67].

Many studies have shown that licochalcone A was proved to perform anti-inflammatory activity. In acute lung injury models, it suppressed activation of NF-*κ*B and phosphorylation of p38MAPK and extracellular regulated protein kinases (ERK) [71]. Licochalcone A reduced LPS-induced NF-*κ*B transactivation by inhibiting phosphorylation of p65 at serine 276 and interaction of p65 with p300 [72]. The compound not only indicated an anti-inflammatory effect on IL-1*β*-stimulated chondrocytes by activating the Nrf2 signaling pathway [73] but also activated Keap1-Nrf2 signaling to inhibit arthritis by enhancing phosphorylation and expression of p62 at serine 349 [74]. ERK and p38 signaling pathways might play a role in attenuation of allergic airway inflammation by licochalcone A [75].

Several compounds of chalcones were discovered to strongly inhibit nitric oxide (NO), IL-6, and prostaglandin E_2_ (PGE_2_) secretion. On one hand, echinatin, licochalcones A-E, isobavachalcone, morachalcone A, DTM, licoagrochalcone C, and 4-hydroxylonchocarpin have been demonstrated to show significant inhibitory activity on LPS-induced NO production [10, 21, 69, 70, 78, 80]. On the other hand, these compounds also have other different anti-inflammatory mechanisms. Licochalcones A and B, echinatin, and DTM inhibited the production of IL-6 and PGE_2_ in LPS-induced macrophage cells. Furthermore, licochalcones B and D reduced LPS-induced production of TNF-*α* and monocyte chemotactic protein 1 (MCP-1) [10, 69, 70].

Licochalcone C played a vital role in sepsis-induced inflammation through repressing NF-*κ*B translocation and several kinds of downstream molecules, including iNOS, intercellular adhesion molecule-1 (ICAM-1), as well as vascular cell adhesion molecule-1 (VCAM-1). Meanwhile, it might upregulate the PI3K/Akt/eNOS signaling pathway [76]. Similar to licochalcone C, licochalcone E reduced expression of inducible enzymes (iNOS and COX-2) and proinflammatory cytokines via the suppression of NF-*κ*B and AP-1 transcriptional activity [77]. Isobavachalcone and kanzonol B suppressed the production of PGE_2_ and NO and downregulated the expression of iNOS and COX-2 through suppressing I*κ*B*α* degradation in LPS-activated microglia [79]. Histone deacetylase enzymes (HDACs), potential drug targets for natural chalcones, were employed to treat cancer and inflammation. Isoliquiritigenin and homobutein might suppress both NF-*κ*B and HDAC activities [68]. Through inhibiting the binding of LPS to TLR4 on immune cells and increasing the polarization of M1 macrophages to M2 macrophages, 4-hydroxylonchocarpin attenuated colitis in mice [81]. The anti-inflammatory mechanism of morachalcone A was related to the activation of MAPKs (p38, ERK, and JNK) and NF-*κ*B pathways, particularly decrease of nuclear translocation of NF-*κ*B p65 subunit [80]. Kanzonol B, DTM, and licoagrochalcone C revealed efficacious inhibitory activity on NF-*κ*B transcription [10].

### 3.3. Antibacterial Activity

There are many antibacterial drugs in the market today. However, with the abuse of antibacterial drugs, the resistance of bacteria is increasing. Therefore, it is crucial to find the new, safe, and effective antibacterial drugs. Some compounds from licorice were proved to have antibacterial activity.

On the basis of previous research studies, isoliquiritigenin had obvious inhibitory effect on methicillin-resistant *Staphylococcus aureus* and *Ralstonia solanacearum* [82, 83]. Licochalcone A, as one of the vital antibacterial components in licorice, was resistant to both bacterial and fungal infections [118, 119]. The study also showed promising antifungal activity of licochalcone A against *Trichophyton rubrum* via inhibiting important antifungal targets of ergosterol synthesis, cell wall synthesis, and glyoxylate cycle [119]. It has been reported to display good activity against *Staphylococcus aureus* biofilm [84]. Licochalcone A exhibited *in vitro* inhibitory effect on human pathogenic *Mycobacteria* species (*Mycobacterium tuberculosis*, *M. bovis*, *M. kansasii*, *M. xenophii*, and *M. marinum*) and *Legionella* species (*Legionella bozemanni*, *L. dumoffii*, *L. feelei*, *L. longbeacheae*, *L. wadsworthii*, *L. gormanii*, and *L. micdadei*), which might be a candidate for treating severe lung infections [85]. Besides, licochalcone A revealed the effects of resisting to all Gram-positive bacteria tested, including spore-forming bacteria, such as genera *Bacillus* and *Clostridium*, and toxin-producing bacteria, such as *Bacillus cereus* and *Ralstonia aureus* [118]. And it affected the growth of *Streptococcus suis* by inhibiting biofilm formation and hemolysin lyase secretion [86]. Licochalcone A also inhibited the growth of *Helicobacter pylori* [87]. Furthermore, it was an effective antifungal agent that might act in synergy with nystatin to inhibit the growth of *Candida albicans* so as to treat candidiasis [88].

A study investigated effects of isobavachalcone, kanzonol C, and 4-hydroxylonchocarpin on 22 strains of microbial species, such as Gram-positive bacteria (*Streptococcus faecalis*, *Staphylococcus aureus*, *Bacillus cereus*, *Bacillus megaterium*, *Bacillus stearothermophilus*, and *Bacillus subtilis*), Gram-negative bacteria (*Citrobacter freundii*, *Enterobacter aerogenes*, *Enterobacter cloacae*, *Escherichia coli*, *Klebsiella pneumonia*, *Morganella morganii*, *Proteus mirabilis*, *Proteus vulgaris*, *Pseudomonas aeruginosa*, *Shigella dysenteriae*, *Shigella flexneri*, and *Salmonella typhi*) and fungi (*Candida albicans*, *Candida glabrata*, *Microsporum auditorium*, and *Trichophyton rubrum*) [89]. Isobavachalcone and kanzonol C could prevent the growth of all the 22 tested microbial species, and 4-hydroxylonchocarpin might suppress the growth of six species of Gram-positive bacteria, four species of fungi, and seven species of Gram-negative bacteria [89]. Isobavachalcone, kanzonol C, and 4-hydroxylonchocarpin also inhibited the reverse transcriptase activity and revealed the antimycobacterial activity against *Mycobacterium tuberculosis* H_37_Rv [90, 120]. Isobavachalcone also showed inhibitory activity against *Candida albicans* and *Candida neoformans* [91].

### 3.4. Antiviral Activity

Licorice is a universal CMM from several prescriptions, which has been proved to inhibit viral infection [121]. At present, there are some antiviral studies on the chalcone compounds from licorice. The components studied include isoliquiritigenin, licochalcone A, isobavachalcone, echinatin, and kanzonol Y. Isoliquiritigenin and licochalcone A evinced anti-HCV activity [92, 93]. Isobavachalcone had potent antiporcine reproductive and respiratory syndrome virus (PRRSV) activity *in vitro* by inhibiting PRRSV replication at the postentry stage of PRRSV infection [94]. Echinatin and isoliquiritigenin showed strong inhibitory effects on influenza viral strains, H1N1, H9N2, and novel H1N1 [28]. Kanzonol Y had antidengue virus (DENV) activity [95].

### 3.5. Antioxidative Activity

Excessive free radicals will accelerate human aging and bring on other diseases [122]. Therefore, enhancing the body's antioxidant capacity is critical to human health. The phenolic hydroxyl structure of chalcones is a good proton donor, which can terminate the oxidative damage by combining with a radical [123]. There are a few chalcone compounds in licorice with antioxidant capacity.

Isoliquiritigenin, as an abundant and forceful antioxidant toward low-density lipoprotein (LDL) oxidation, could attenuate atherosclerosis [96]. Isoliquiritigenin and paratocarpin B were found to be the most powerful antioxidant agents by an authentic peroxynitrite antioxidant assay [26]. Licochalcones A, B, and D, DTM, and isobavachalcone inhibited NADPH-induced microsomal lipid peroxidation [10, 22]. Isobavachalcone also suppressed ascorbate-, *t*-BuOOH-, and CCl_4_-induced microsomal lipid peroxidation, and NADH-dependent and ascorbate-induced mitochondrial lipid peroxidation [100]. Studies manifested that echinatin and licochalcone B possessed the strong scavenging activity toward ABTS^*·*+^ radical [10]. Licochalcones B and D strongly suppressed superoxide anion production and expressed powerful scavenging activity on DPPH radical [22]. Licochalcone A protected skin cells from oxidative stress by activating Nrf2-signaling, resulting in increased expression of HO-1 and glutamate-cysteine ligase regulatory subunit (GCLM) and reduction of intracellular ROS concentration [97]. Moreover, licochalcones A and C increased the expression of antioxidant enzymes including superoxide dismutase (SOD), catalase (CAT), and glutathione peroxidase (GPx) proteins that were closely related to antioxidant mechanisms [98, 99]. Glypallichalcone, the inhibitor of LDL oxidation, reduced cholesterol levels through modulation of *β*-1 adrenergic receptor [101]. Dihydroisoliquiritigenin was used as a neuroprotectant against glutamate-induced oxidative stress in a mouse-derived hippocampal neuronal cell line (HT22) [102].

### 3.6. Hepatoprotective Activity

Liver is a vital organ that performs many metabolic functions in the body such as detoxification, glycogen storage, and protein synthesis [124]. CCl_4_ is a chemical substance that is severely toxic to hepatocytes [125]. Also, overuse of acetaminophen (APAP) could cause drug-induced liver toxicity [126]. The natural ingredients of CMMs are considered to be an effective and safe alternative way to treat hepatocyte damage [11]. A few chalcone constituents in licorice have been proved to possess significant hepatoprotective activity.

For example, echinatin and licochalcones A and B showed Nrf2 activation activities on HepG2C8 cells in ARE-luciferase reporter assay and a promising hepatoprotective effect on CCl_4_-induced acute liver injury in the ICR mice model [104]. Additionally, isoliquiritigenin, licochalcone B, and licoagrochalcone A possessed a remarkably potent protective effect against both CCl_4_- and APAP-induced HepG2 cell injuries. 3,4,3′,4′-Tetrahydroxychalcone also showed significantly potent activity against CCl_4_-induced liver injury [103]. Moreover, studies have demonstrated that licochalcone B might protect hepatocytes from alcohol-induced cell damage by reducing apoptosis, inhibiting oxidative stress, and upregulating Erk-Nrf2 [105]. Licochalcone E basically exhibited its protective role for treating hepatotoxicity through the peroxisome proliferator-activated receptor-*γ* (PPAR-*γ*)/NF-*κ*B-mediated pathway [106].

### 3.7. Antidiabetic Activity

Diabetes is a group of metabolic diseases characterized by hyperglycemia [127]. Long-standing hyperglycemia in diabetes leads to various tissue dysfunctions [128]. At present, drugs for the treatment of this disease are urgently needed to be developed. As confirmed by the experiment, extracts from licorice have a protective effect on diabetic nephropathy [129].

Through the oral glucose tolerance test, the hypoglycemic effect of isoliquiritigenin on normal Swiss albino male mice was reported [107]. Another finding demonstrated that isoliquiritigenin diminished high glucose-induced mesangial matrix accumulation through retarding transforming growth factor (TGF)-*β*1-SMAD signaling transduction [108]. Angiotensin-converting enzyme (ACE) plays a prominent role in hypertension, heart failures, myocardial infarction, and diabetic nephropathy. The study showed that echinatin has been proved to show a certain inhibitory effect on ACE *in vitro* [109]. Furthermore, licochalcone E enhanced expression of PPAR-*γ* through irritating Akt signals as well as functions as a PPAR-*γ* partial agonist, which improved hyperlipidemia and hyperglycemia under diabetic conditions [12]. Kanzonol C, licoagrochalcone A, and isobavachalcone as inhibitors of protein tyrosine phosphate 1B (PTP1B) were potential candidates for treating type II diabetes [21, 110].

### 3.8. Antiobesity Activity

Obesity is a globally epidemic chronic metabolic disease, and the proportion of obese people continues to rise due to changes in lifestyle and diet. Obesity poses a series of potential safety hazard, so the use of antiobesity drugs can help improve the health of patients. Kanzonol C, licoagrochalcone A, and isobavachalcone were found to be PTP1B inhibitors for treatment of obesity [21, 110]. Isoliquiritin apioside and isoliquiritigenin, as sources of pancreatic lipase (PL) inhibitors for preventing obesity, could lower the plasma total triglycerides and total cholesterol [111]. Licochalcone A had an inhibitory effect on adipocyte differentiation and lipogenesis via the downregulation of PPAR-*γ*, CCAAT/enhancer binding protein *α* (C/EBP*α*), and sterol regulatory element-binding protein 1c (SREBP-1c) in 3T3-L1 preadipocytes [112]. And other results demonstrated that licochalcone A was effective to reduce obesity and could recover metabolic homeostasis by inducing adipocyte browning [113].

### 3.9. Other Activities

Isoliquiritigenin has been detected to have antiplatelet action [130], protective effect on cerebral ischemia injury [131], and estrogen-like [132], neuroprotective [133], and antimelanogenic [134] activities. Licochalcone A has been demonstrated to possess antispasmodic [135], antileishmanial [136], antimalarial [137], and osteogenic activities [138]. Isoliquiritin was studied to produce significant antidepressant-like effect [13].

## 4. Conclusion

Phytochemical constituents especially flavonoids are largely considered to be beneficial for human health and disease prevention. As a category of nontoxic and effective natural ingredients, chalcones are proved to possess lots of biological activities and medicinal properties. To date, about 42 chalcones in licorice have been isolated and identified, and more new structures will be unveiled. Meanwhile, most of chalcones in licorice have been widely and deeply studied for their various activities, such as anticancer, anti-inflammatory, antimicrobial, antiviral, antioxidative, hepatoprotective, antidiabetic, and antiobesity activities. However, it will be a long way to further validate the pharmacological action and develop new drug. As chalcones in licorice are deeply explored and fully utilized, it will be served as a broad prospect for development and utilization of licorice.

## Figures and Tables

**Table 1:** The sources of chalcones from licorice.

Number	Name	Source	Reference
C1	Isoliquiritin apioside	*G. glabra* L. *G. uralensis* Fisch. *G. inflata* Bat.	[16, 17]
C2	Licuraside	*G. glabra* L. *G. uralensis* Fisch. *G. inflata* Bat.	[17]
C3	Isoliquiritin	*G. glabra* L. *G. uralensis* Fisch. *G. inflata* Bat.	[16–18]
C4	Butein-4-*O*-*β*-D-glucopyranoside	*G. uralensis* Fisch.	[19]
C5	Neoisoliquiritin	*G. glabra* L. *G. uralensis* Fisch. *G. inflata* Bat.	[14, 16, 20]
C6	Isoliquiritigenin	*G. glabra* L. *G. uralensis* Fisch. *G. inflata* Bat.	[16, 17, 21–24]
C7	Homobutein	*G. uralensis* Fisch.	[18]
C8	Echinatin	*G. uralensis* Fisch. *G. glabra* L. *G. inflata* Bat.	[10, 18, 21, 22, 25]
C9	Licochalcone A	*G. glabra* L. *G. uralensis* Fisch. *G. inflata* Bat.	[10, 14, 17]
C10	Licochalcone B	*G. uralensis* Fisch. *G. glabra* L. *G. inflata* Bat.	[10, 21, 22, 25]
C11	5-(1,1-Dimethylallyl)-3,4,4′-trihydroxy-2-methoxychalcone (DTM)	*G. uralensis* Fisch. *G. inflata* Bat.	[10, 21]
C12	Licochalcone C	*G. glabra* L. *G. inflata* Bat.	[21, 22, 26]
C13	Licochalcone D	*G. inflata* Bat.	[22]
C14	Licochalcone E	*G. inflata* Bat.	[21, 22]
C15	Isobavachalcone	*G. glabra* L. *G. inflata* Bat.	[21, 27]
C16	Kanzonol C	*G. inflata* Bat.	[21]
C17	Corylifol B	*G. inflata* Bat.	[21]
C18	Morachalcone A	*G. glabra* L.	[25]
C19	2,3′,4,4′-Tetrahydroxy-3,5′-diprenylchalcone	*G. glabra* L.	[25]
C20	Kanzonol B	*G. glabra* L. *G. inflata* Bat.	[21, 27]
C21	3,3′,4,4′-Tetrahydroxy-2′-methoxy-5-prenylchalcone	*G. glabra* L.	[25]
C22	Licochalcone G	*G. glabra* L.	[28]
C23	2′,3,4′-Trihydroxy-3′-*γ*,*γ*-dimethylallyl-6″,6″-dimethylpyrano[2″,3″:4,5] chalcone	*G. glabra* L.	[25]
C24	3,4,3′,4′-Tetrahydroxychalcone	*G. inflata* Bat.	[21]
C25	3,4,3′,4′-Tetrahydroxy-2-methoxychalcone	*G. glabra* L.	[16]
C26	Licoagrochalcone A	*G. glabra* L. *G. inflata* Bat.	[21, 27]
C27	Licoagrochalcone B	*G. glabra* L.	[26]
C28	Licoagrochalcone C	*G. glabra* L. *G. inflata* Bat.	[21, 26]
C29	Licoagrochalcone D	*G. glabra* L.	[26]
C30	Glypallichalcone	*G. glabra* L.	[29]
C31	Paratocarpin A	*G. glabra* L.	[23]
C32	Paratocarpin B	*G. glabra* L.	[23]
C33	4-Hydroxylonchocarpin	*G. glabra* L.	[27]
C34	Dihydroisoliquiritigenin	*G. inflate* Bat.	[24]
C35	Glycybridin A	*G. glabra* L.	[30]
C36	Kanzonol Y	*G. glabra* L.	[26]
C37	1-(2′,4′-Dihydroxyphenyl)-2-hydroxy-3-(4″-hydroxyphenyl)-1-propanone	*G. glabra* L.	[25]
C38	2,3′,4,4′,*α*-Pentahydroxy-3,5′-diprenyl-dihydrochalcone	*G. glabra* L.	[25]
C39	2,3′,4,4′,*α*-Pentahydroxy-3-prenyl-dihydrochalcone	*G. glabra* L.	[25]
C40	Glycybridin B	*G. glabra* L.	[30]
C41	Glycybridin C	*G. glabra* L.	[25, 30]
C42	2′,4′-Dihydroxydihydrochalcone-4-*O*-*β*-D-glucopyranoside	*G. uralensis* Fisch.	[19]

**Table 2:** The biological activities of chalcones from licorice.

Biological activity	Compounds	Reference
Anticancer	Isoliquiritigenin	[31–35]
	Isoliquiritin	[32]
	Licochalcone A	[36–42]
	Licochalcone B	[43, 44]
	Licochalcone C	[45, 46]
	Licochalcone D	[45, 46]
	Licochalcone E	[47–49]
	Kanzonol C	[50]
	Isobavachalcone	[51]
	Echinatin	[52, 53]
	Isoliquiritin apioside	[54]
	Paratocarpin A	[55]
	4-Hydroxylonchocarpin	[56, 57]
	Kanzonol Y	[58]

Anti-inflammatory	Isoliquiritin	[59]
	Isoliquiritigenin	[59–68]
	Homobutein	[68]
	Echinatin	[10, 69, 70]
	Licochalcone A	[10, 70–75]
	Licochalcone B	[10, 21, 69, 70]
	Licochalcone C	[21, 69, 76, 77]
	Licochalcone D	[69, 70]
	Licochalcone E	[21, 77]
	Isobavachalcone	[78, 79]
	Morachalcone A	[80]
	Kanzonol B	[10, 79]
	DTM	[10, 21]
	Licoagrochalcone C	[10, 21]
	4-Hydroxylonchocarpin	[78, 81]

Antibacterial	Isoliquiritigenin	[82, 83]
	Licochalcone A	[84–88]
	Isobavachalcone	[89–91]
	Kanzonol C	[89, 90]
	4-Hydroxylonchocarpin	[89]

Antiviral	Isoliquiritigenin	[28, 92]
	Licochalcone A	[92, 93]
	Isobavachalcone	[94]
	Echinatin	[28]
	Kanzonol Y	[95]

Antioxidative	Isoliquiritigenin	[26, 96]
	Licochalcone A	[97, 98]
	Licochalcone B	[10, 22]
	Licochalcone C	[99]
	Licochalcone D	[22]
	Isobavachalcone	[100]
	Echinatin	[10]
	DTM	[10]
	Paratocarpin B	[26]
	Glypallichalcone	[101]
	Dihydroisoliquiritigenin	[102]

Hepatoprotective	Isoliquiritigenin	[103]
	Echinatin	[104]
	Licochalcone A	[104]
	Licochalcone B	[103–105]
	Licochalcone E	[106]
	Licoagrochalcone A	[103]
	3,4,3′,4′-Tetrahydroxychalcone	[103]

Antidiabetic	Isoliquiritigenin	[107, 108]
	Licochalcone E	[12]
	Echinatin	[109]
	Isobavachalcone	[110]
	Kanzonol C	[21]
	Licoagrochalcone A	[21]

Antiobesity	Kanzonol C	[21]
	Licoagrochalcone A	[21]
	Isobavachalcone	[110]
	Isoliquiritin apioside	[111]
	Isoliquiritigenin	[111]
	Licochalcone A	[112, 113]
